# miR-20a suppresses chondrogenic differentiation of ATDC5 cells by regulating Atg7

**DOI:** 10.1038/s41598-019-45502-7

**Published:** 2019-06-25

**Authors:** Rui Xu, Yuhao Wei, Xing Yin, Bing Shi, Jingtao Li

**Affiliations:** 1 State Key Laboratory of Oral Diseases & National Clinical Research Centre for Oral Diseases & Department of Oral and Maxillofacial Surgery, West China Hospital of Stomatology, Sichuan University, 14 Ren Min Nan Road, Chengdu, 610041 P.R. China; 2 State Key Laboratory of Oral Diseases & National Clinical Research Centre for Oral Diseases & Department of Orthodontics, West China Hospital of Stomatology, Sichuan University, 14 Ren Min Nan Road, Chengdu, 610041 P.R. China

**Keywords:** Macroautophagy, Bone development, Cartilage development

## Abstract

Both the miR-17-92 cluster and autophagy have been suggested as critical regulators of bone development, but the potential correlation between the two factors is largely unknown. Hence, we investigated whether members of this cluster can regulate chondrogenesis through an autophagy-related signalling pathway. In this study, the expression of miR-17-92 cluster members and the level of autophagic activity were investigated during chondrogenic induction in ATDC5 cells. miR-17, miR-18a, miR-20a, and miR-92-1 showed significant changes, and the level of autophagic activity was enhanced. Among the miR-17-92 cluster members, miR-20a showed the most significant change. Histological, cellular and molecular analyses were performed after the regulation of miR-20a and autophagy. miR-20a and autophagy had the opposite effect on chondrogenic differentiation, and there was a negative correlation between them. Moreover, the expression of the autophagy regulatory gene Atg7 was inhibited by miR-20a. siRNA was then used to knock down Atg7, and the results further indicated that Atg7 might be a potential target of miR-20a in chondrogenic differentiation. In conclusion, miR-20a is a critical negative regulator of chondrogenic differentiation because it inhibits autophagy via Atg7. Other members of the miR-17-92 cluster may have a similar effect, but this hypothesis requires further investigation.

## Introduction

Autophagy is acknowledged to play an important role in development and diseases. This process is an evolutionarily conserved intracellular catabolic mechanism that recycles nutrients and energy through the degradation of macromolecules and organelles by lysosomes^1^. Autophagy involves the formation of autophagosomes, phagocytosis of macromolecules and organelles, transfer of autophagosomes into lysosomes, and degradation in the autolysosomes. Generally, autophagy occurs at a low basal level, and it is rapidly upregulated when cells need to generate intracellular nutrients and energy in response to diverse stress conditions. Deregulation of autophagy has been associated with developmental disorders, bone and cartilage diseases, diseases of other organs and tissues, such as the liver, heart, muscle and nerves, and cancers^2,3^.

Recently, the role of autophagy in the physiology of cartilage has been intensively studied. FGF signalling, which is essential in chondrogenesis, has been reported to regulate cartilage development by targeting the conjugation of Atg5/Atg12^4^. Lack of Atg7 results in the dysfunction of type II collagen (col2) synthesis and secretion^5^. Moreover, autophagy promotes the survival of hypertrophic chondrocytes in growth plates^6^ and prevents damage from physically or chemically induced osteoarthritis in articular cartilage^7–9^.

Accumulating data have correlated autophagy and the functions of microRNAs (miRNAs)^5,10–12^. miRNAs are small non-coding RNA molecules of approximately 22 nucleotides that function in RNA silencing and the post-transcriptional regulation of eukaryotic gene expression. By binding to the 3′-UTR of target mRNAs, miRNAs can inhibit protein translation or result in mRNA degradation. In this way, they are widely involved in physiological processes such as development, tumourigenesis, cell proliferation and apoptosis^13^.

The miR-17-92 cluster is expressed as a polycistronic primary transcript containing six tandem stem-loop hairpins, and it ultimately results in six mature miRNAs (miR-17, miR-18a, miR-19a, miR-20a, miR-19b, and miR-92a). This cluster plays pivotal roles in regulating cellular processes, including proliferation, apoptosis, inflammatory activation and autophagy^14–18^. miR-20a is reported to target Atg7 and Atg16L and promotes mycobacterial survival in macrophage cells^19^. miR-18a and miR-92a have been reported to regulate chondrogenesis, but the role of this cluster in chondrogenic differentiation requires further exploration^20–23^.

Considering that members of the same cluster may have synergistic effects and that signalling crosstalk may occur at autophagy-related proteins (ATGs), we speculate that the miR-17-92 cluster can regulate chondrogenic differentiation or chondrogenesis by targeting downstream ATGs such as Atg7.

## Results

### Expression of the miR-17-92 cluster decreases during ATDC5 chondrogenic differentiation

**Figure 1:**
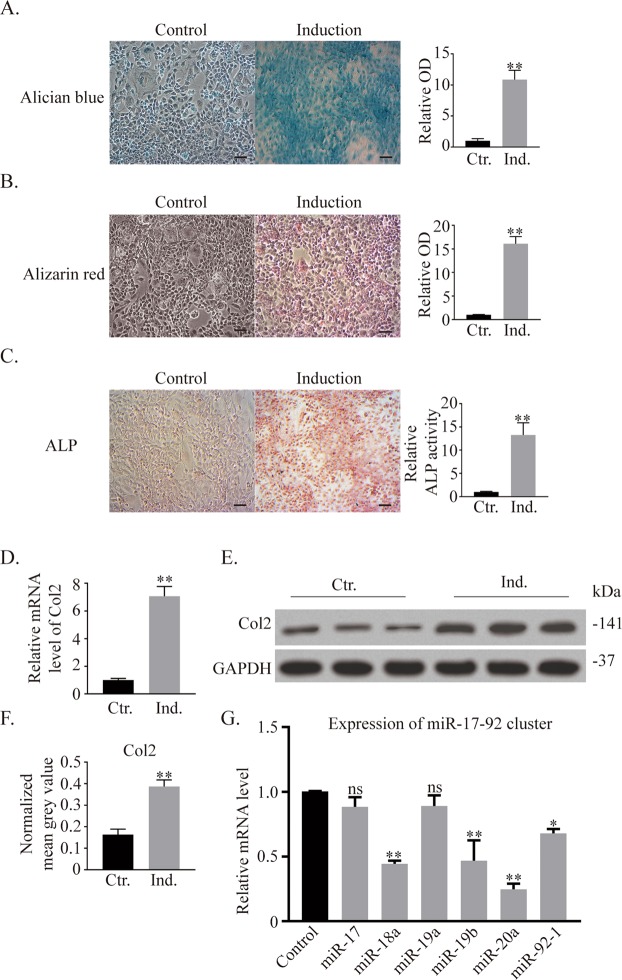
Expression of miR-17-92 cluster members decreases during ATDC5 chondrogenic differentiation. (**A**–**C**) Alcian blue, alizarin red and ALP staining of ATDC5 cells. Scale bar = 100 μm. (**D**) mRNA expression of col2 analysed by PCR. (**E,F**) Western blotting results of col2 after culture. Signal intensities were quantified using ImageJ software (version 1.80) (**G**) Expression of miR-17-92 cluster members during ATDC5 chondrogenic differentiation. *ALP* = *alkaline phosphatase, col2* = collagen type-II; the error bars indicate the standard deviation, *p < 0.05, **p < *0.01, ns: not significant*.

As the miR-17-92 cluster plays an important role in cell proliferation and differentiation in various cells, such as cardiomyocytes^24^, lung epithelial cells^25^ and palatal mesenchymal cells^26^, we questioned whether the miR-17-92 cluster is involved in cellular chondrogenic differentiation. After ten days of chondrogenic induction, ATDC5 cells demonstrated higher levels of proteoglycan expression (Fig. 1A), mineralization (Fig. 1B) and alkaline phosphatase activity (Fig. 1C) than control cells, as evidenced by the mRNA and protein expression of col2 (Fig. 1D–F). All six members of the miR-17-92 cluster invariably decreased during induction (Fig. 1G), suggesting a negative correlation between the miR-17-92 cluster and chondrogenesis. Specifically, the decreases in miR-18a, miR-19b, miR-20a, and miR-92-1 were statistically significant. miR-20a demonstrated the largest decrease, highlighting its potential effect on chondrogenic differentiation.

### miR-20a suppresses ATDC5 chondrogenic differentiation

**Figure 2:**
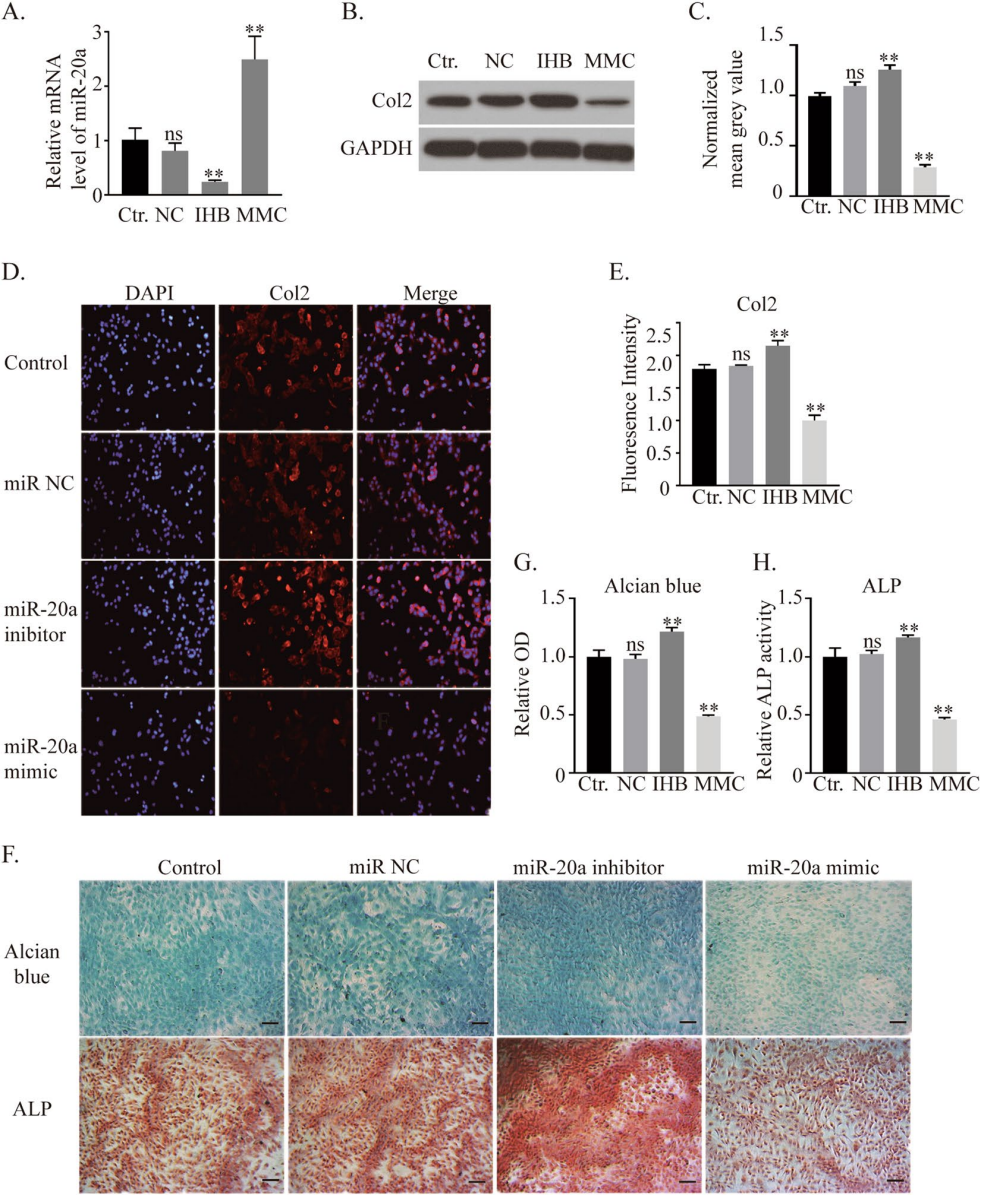
Regulation of miR-20a affects the chondrogenic differentiation of ATDC5 cells. (**A**) Expression of miR-20a after miRNA negative control, miR-20a inhibitor and miR-20a mimic transfection. (**B,C**) Western blot results of col2 and its quantification. (**D,E**) Immunohistochemistry analysis of col2 after regulating the expression of miR-20a. (**F**–**H**) Alcian blue and ALP staining and quantitative analysis. Scale bar = 100 μm. *Ctr*. = *control, NC* = *miRNA negative control, IHB* = *miR-20a inhibitor, MMC* = miR-20a mimic. *p < 0.05, **p < *0.01, ns: not significant*.

To test whether miR-20a inhibits chondrogenic differentiation, a miR-20a mimic, miR-20a inhibitor and miRNA NC (negative control) were transfected into ATDC5 cells. qRT-PCR analyses demonstrated miR-20a suppression in the miR-20a inhibitor group and miR-20a overexpression in the miR-20a mimic group, indicating successful transfection (Fig. 2A). According to the western blot results, the expression of col2 was not significantly different between the miRNA NC group and the control group, but it was significantly decreased by 72% in the miR-20a mimic group and significantly increased by 28% in the miR-20a inhibitor group (Fig. 2B,C). A col2 immunofluorescent assay showed similar results (Fig. 2D,E). Alcian blue and ALP histochemical staining (Fig. 2F–H) also demonstrated higher proteoglycan levels and alkaline phosphatase activity in the groups with less miR-20a. These data suggested that miR-20a inhibited chondrogenic differentiation in ATDC5 cells.

### Autophagy is enhanced during ATDC5 chondrogenic differentiation

**Figure 3:**
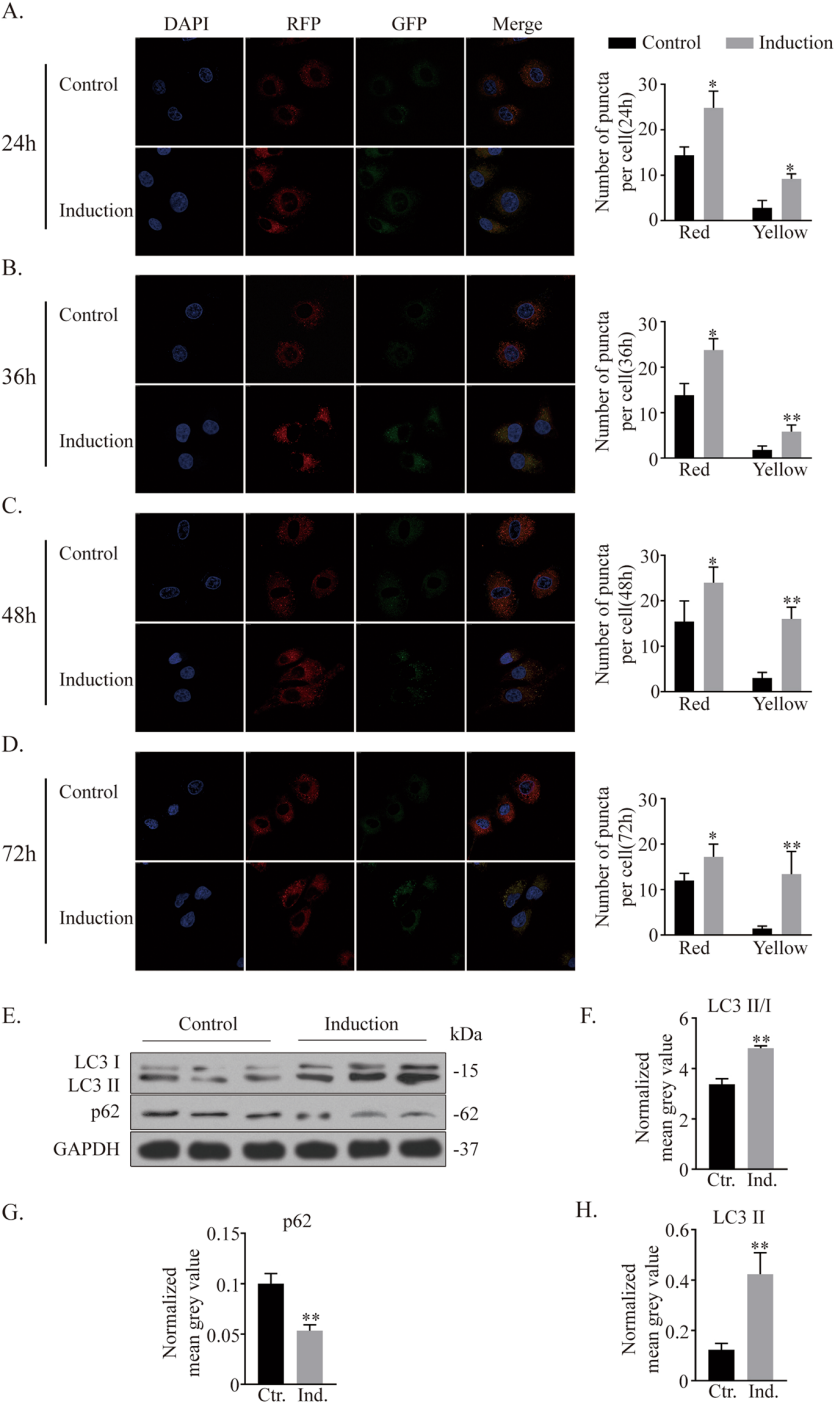
Autophagy is activated during the chondrogenic differentiation of ATDC5 cells. (**A**–**D**) Images taken at 24 h, 36 h, 48 h, and 72 h to monitor autophagic flux. Autophagic activity is reflected by the quantification of autophagic puncta. (**E**–**H**) Western blotting results of autophagy-related proteins after chondrogenic induction and quantitative analysis using ImageJ software. *p < 0.05, **p < *0.01*.

To investigate the level of autophagy during chondrogenic differentiation, ATDC5 cells were transfected with an mRFP-GFP-LC3 adenoviral vector, and autophagic flux was measured at multiple time points because autophagy is a dynamic process (Fig. 3A–D). At all the time points, the numbers of both red and yellow puncta significantly increased in the induction group, indicating that autophagic activity was higher in the chondrogenic induction group^27^.

Western blot results showed evident increases in both LC3-I and LC3-II (with increased LC3-I/LC3-II conversion) and decreases in the autophagosome cargo protein p62 after ten days of induction culture compared to control conditions (Fig. 3E–H). These data suggested that autophagy was enhanced during ATDC5 cell chondrogenic differentiation.

### miR-20a suppresses autophagy during ATDC5 cell chondrogenic differentiation

**Figure 4:**
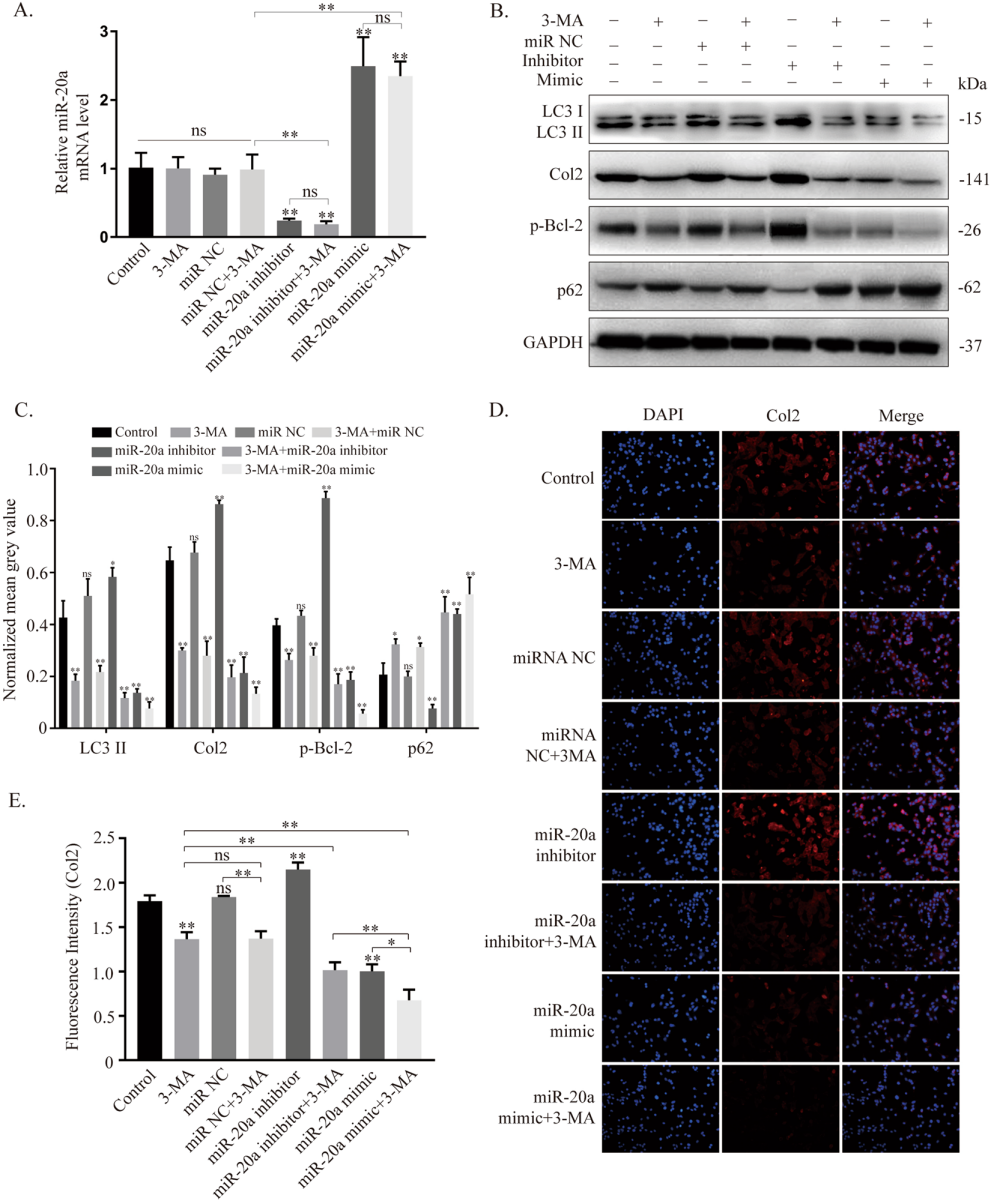
miR-20a inhibits autophagy activity during chondrogenic differentiation. (**A**) mRNA expression of miR-20a in eight groups after transfection of miRNA reagents, addition of an autophagy inhibitor or their combination under inducing culture. (**B,C**) Western blotting results of autophagy-related proteins, including LC3-II/I, p-Bcl-2, and p62, and the chondrogenic marker protein col2. Comparisons between each of the two groups were analysed using Dunnett’s test. (**D,E**) Immunofluorescent assay of col2 and quantification by ImageJ. *p < 0.05, **p < *0.01*. *Without a connecting line is compared to the control group.

The potential correlation between miR-20a and autophagy was further tested by exogenous delivery of miR-20a inhibitor/mimic and an autophagy inhibitor (3-MA). PCR analyses verified the effectiveness of both the miR-20a inhibitor and mimic in manipulating miR-20a expression (Fig. 4A). No significant difference in the expression of miR-20a was detected among the control, 3-MA, miRNA NC and miRNA NC + 3-MA groups. Likewise, there was no significant difference in miR-20a expression between the miR-20a inhibitor group and the miR-20a inhibitor + 3-MA group and between the miR-20a mimic group and the miR-20a mimic + 3-MA group, indicating that autophagy inhibition by 3-MA did not influence the expression of miR-20a.

**Figure 5:**
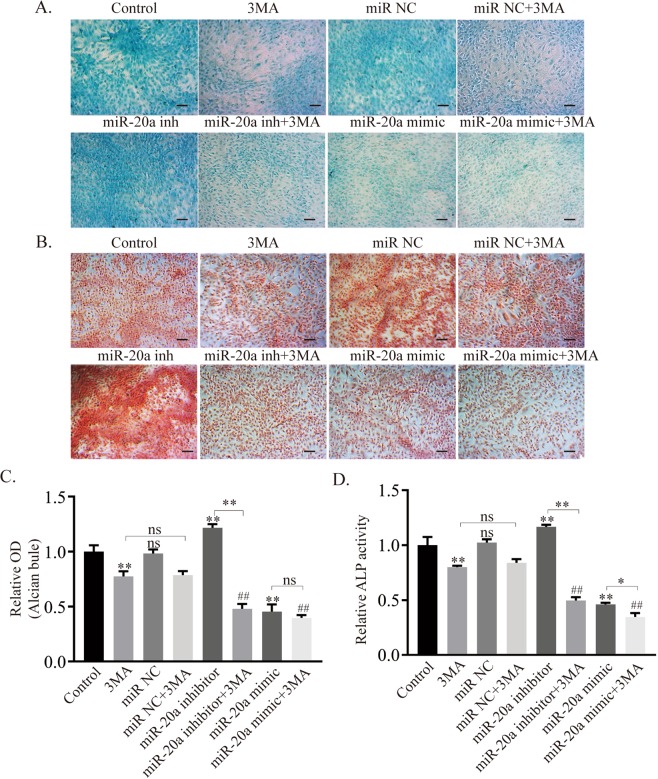
Increased expression of miR-20a and inhibition of autophagy impair the chondrogenic process of ATDC5 cells. (**A,C**) Alcian blue staining and quantification. (**B,D**) ALP staining and quantification. Scale bar = 100 μm, *p < 0.05, **p < *0.01*, *Without a connecting line is compared to the control group, ## is compared to the 3-MA group.

At the protein level (Fig. 4B,C), the expression of LC3-II/I, p-Bcl-2 and col2 decreased, while that of p62 increased in the groups treated with 3-MA or miR-20a mimic. The addition of 3-MA and the miR-20a inhibitor induced a dramatic shift from autophagy activation to autophagy suppression. When 3-MA was added to the miR-20a mimic group, the suppression of autophagy was aggravated. The immunofluorescent results for col2 showed that its expression was positively correlated with the autophagy level and negatively correlated with miR-20a expression (Fig. 4D,E). At the same time, alcian blue and ALP (Fig. 5A–D) staining showed that the synthesis of proteoglycans and the activity of alkaline phosphatase were inhibited under the conditions of miR-20a overexpression and autophagy suppression.

Collectively, miR-20a suppressed the level of autophagy during the chondrogenic differentiation of ATDC5 cells.

### miR-20a suppresses autophagy by inhibiting Atg7 during chondrogenic differentiation

Since miR-20a was reported to target Atg7^19,28^, which has been associated with col2 synthesis and secretion and cartilage development^4,5^, we questioned whether miR-20a inhibited autophagy via Atg7 during chondrogenic differentiation. Hence, Atg7 siRNA was introduced into the *in vitro* culture system to explore this mechanism.

**Figure 6:**
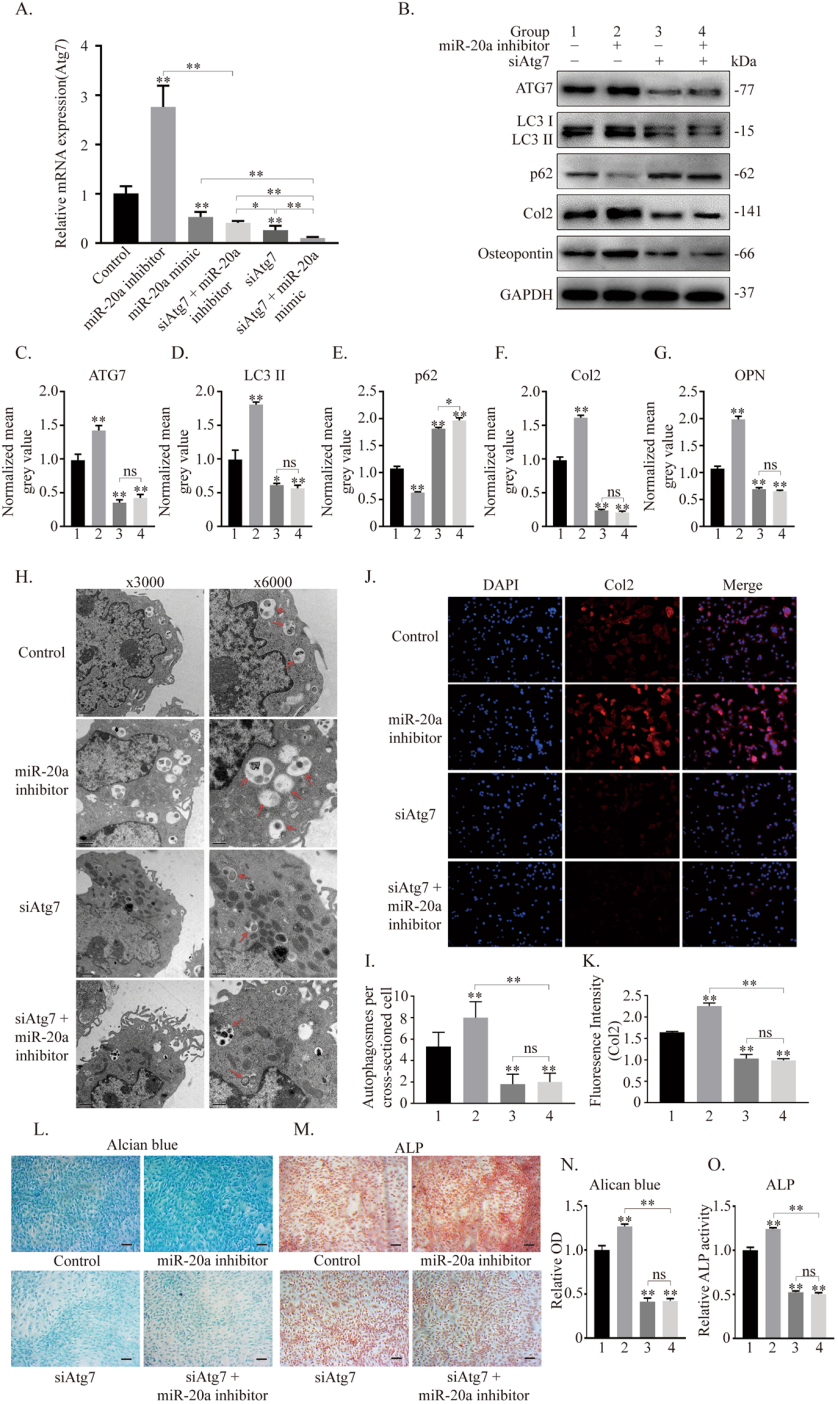
miR-20a suppresses autophagy by downregulating Atg7 during the chondrogenic process of ATDC5 cells. (**A**) mRNA expression of Atg7. (**B–G**) Expression of ATG7, LC3-I, LC3-II, p62, col2 and osteopontin evaluated by western blotting and analysed according to the mean grey value. (**H,I**) TEM analysis of autophagic vesicles within ATDC5 cells. The red arrow indicates autophagic vesicles, including autophagosomes and autolysosomes. Magnification: 3000×, 1 μm; and 6000×, 0.5 μm for close-up images. The numbers of autophagic puncta were counted per cross-sectioned cell and compared using Student’s t-test. (**J,K**) Immunofluorescent assay of col2 among groups and quantification of fluorescence intensity. (**L–O**) Histochemical alcian blue and ALP staining and corresponding quantitative analysis. Scale bar = 100 μm. *p < 0.05, **p < *0.01*.

According to PCR analyses, the expression of Atg7 was found to be significantly upregulated when miR-20a was inhibited and significantly downregulated when miR-20a was overexpressed. Transfection of siAtg7 effectively inhibited Atg7 expression in ATDC5 cells (Fig. 6A). The expression level of ATGs, including Atg7, p62 and LC3 conversion, significantly increased in the miR-20a inhibitor group but decreased in the groups treated with siAtg7. The protein expression levels of col2 and osteopontin were basically in accordance with the level of autophagy (Fig. 6B–G). The number of autophagic vacuoles was detected by transmission electron microscopy (TEM). This number was increased in the miR-20a inhibitor group but decreased in the siAtg7 groups when compared to the that in the control group. Moreover, there was no significant difference between the other two groups (Fig. 6H,I). The immunofluorescent assay showed that the expression of col2 increased in the miR-20a inhibitor group, which supports the suppression of miR-20a. The expression of col2 decreased in the siAtg7 groups, even with miR-20a inhibitor treatment, but there was still no difference between them (Fig. 6J,K). Chondrogenic differentiation was enhanced in the miR-20a inhibitor group and was decreased in the groups treated with siAtg7 as demonstrated by alcian blue and ALP staining (Fig. 6L–O). Taken together, these data suggest that Atg7 is a potential target through which miR-20a suppresses autophagy during chondrogenic differentiation.

## Discussion

Due to their extensive and rapid proliferation, homogeneity and chondrogenic capacity, ATDC5 cells have been recognized as a good model to study chondrogenesis *in vitro*. Evidence has shown that ATDC5 cells have properties that replicate the multiple steps of chondrocyte differentiation. ATDC5 cells undergo cellular condensation and sequential chondrogenic differentiation with characteristic proteoglycan synthesis and col2 expression when treated with insulin^28–30^.

In the miR-17-92 cluster, miR-18a and miR-92a have been correlated with chondrogenesis in previous studies^20–23^. miR-18a suppresses the expression of CCN2/connective tissue growth factor (CCN2/CTGF), causing the suppression of chondrogenic differentiation^20^. miR-92a is highly enriched in chondrogenic progenitors, and its inactivation results in the loss of pharyngeal cartilage elements. miR-92a sustains chondrogenesis through BMP signalling by suppressing noggin3^21^. Moreover, miR-92a directly targets histone deacetylase 2 (HDAC2) to mediate the suppression of cartilage-specific gene expression in human chondrocytes^22^. miR-92a also regulates the expression of aggrecanase-1 and aggrecanase-2 in human chondrocytes and may be involved in the development of osteoarthritis^23^. All of these studies demonstrate the correlation between the miR-17-92 cluster and chondrogenesis. For the first time, our data suggested that miR-20a is another member of the cluster that acts as an important regulator of chondrogenic differentiation. During the chondrogenic differentiation of ATDC5 cells, most members of the miR-17-92 cluster, including miR-18a, miR-19b, miR-20a and miR-92-1, showed a significant decrease, indicating that these four members may all be involved in the regulation of chondrogenic differentiation. This study focused on miR-20a because it had the biggest change. Inhibition and overexpression experiments clarified the potential role of miR-20a in regulating chondrogenic differentiation. The synthesis of col2 and proteoglycans, the activity of alkaline phosphatase, and the extent of mineralization were all enhanced when miR-20a was downregulated. These changes were suppressed when miR-20a was upregulated. However, the extent of increase in chondrogenesis in the miR-20a inhibitor group was not the same as the extent of reduction in the miR-20a mimic group, which indicated a potential synergistic effect from other members of the cluster.

Previous studies have shown differences in the expression of ATGs in chondrogenic differentiation. The expression of beclin1 and LC3 increases during chondrogenic differentiation in adipose stem cells^31^ and decreases during differentiation in mesenchymal stem cells^32^. The difference between these studies may be due to the different cell lineages and observations at different differentiation stages, but the results indicate the role of autophagy in drastic morphological and structural cellular changes. Moreover, multiple studies report that chondrogenic differentiation can be downregulated by decreasing the expression of ATGs^31,33^. Our study confirmed this regulatory role of autophagy in chondrogenic differentiation. In the present study, LC3-II and p-Bcl-2 increased, p62 decreased, and the LC3-II/I ratio increased, indicating enhanced autophagy activity during chondrogenic differentiation in ATDC5 cells. Considering that autophagy is a dynamic process, we employed a tandem monomeric RFP-GFP-tagged LC3 system (mRFP-GFP-LC3) to detect the autophagy flux. The increases in the numbers of yellow puncta and red puncta indicated enhanced autophagy compared to the control group, which supported the western blot results.

The members of the miR-17-92 cluster have been reported to potentially regulate autophagy^34^. The miR-17-92 cluster and its paralogs, including miR-106a-92 and miR-106b-25, share the same seed sequence (AAAGUGC), which is highly expressed in myeloid progenitors, and SQSTM1/p62 has been identified as a target for these miRNAs^35^. miR-20a negatively regulates autophagy by targeting ATG16L1 in hypoxia-induced osteoclast differentiation^36^ and ULK1 in C2C12 myoblasts^37^. Moreover, miR-20a can target Atg7 to suppress tumour proliferation in neuroblastoma^38^ and to promote mycobacterial survival in macrophage cells^19^. These studies confirm that the miR-17-92 cluster can regulate autophagy by targeting various ATGs. In our study, miR-20a upregulation reduced the autophagy level, while miR-20a downregulation increased the autophagy level, indicating that miR-20a had an inhibitory effect on autophagy. The overexpression and inhibition of miR-20a affected the expression of ATGs, while the use of an autophagy inhibitor did not significantly change the expression of miR-20a, implying that the autophagy pathway was downstream of miR-20a and that there was no obvious feedback regulation. The expression of Atg7 significantly increased in the miR-20a inhibitor group and decreased in the miR-20a mimic or siAtg7 groups. After siAtg7 was added, the expression of ATGs and chondrogenic marker proteins in the miR-20a inhibitor group was reversed. Accordingly, the quantification of autophagosomes per cellular cross-section by TEM, immunofluorescent and histochemical analyses supported these results. However, there was no significant difference between the siAtg7 group and the siAtg7 + miR-20a inhibitor group, indicating that the miR-20a inhibitor did not ameliorate the autophagy inhibition caused by siAtg7 and chondrogenic differentiation through other potential targets or pathways. That is, Atg7 is likely to be the target through which miR-20a regulates chondrogenic differentiation in ATDC5 cells.

Because the ATDC5 cell line derives from mouse teratocarcinoma fibroblastic cells, it can provide fundamental biological insights, but it cannot replace normal cell lineages such as MSCs, so it would be better to verify these results in MSCs and ECSs. Due to the incomplete effectiveness of siAtg7, Atg7 expression was knocked down by only 60~70% instead of being completely knocked out, and the regulatory effect of miR-20a on autophagy during chondrogenesis also needs to be verified in knockout mice. 3-MA effectively blocks autophagy at the early stage by inhibiting class III PI3K, but its specificity is questionable. It is suggested that 3-MA also inhibits class I PI3K and can thus promote autophagy at suboptimal concentrations. However, 3-MA does not block beclin1-independent autophagy.

In conclusion, our study suggests that miR-20a is a negative regulator of chondrogenesis by regulating Atg7, which is a possible intervention target for chondrogenic differentiation.

## Materials and Methods

### Cell culture

The murine ATDC5 cell line was purchased from Bang-yi Biotechnology (Shanghai, China) and cultured at 37 °C in a humidified atmosphere of 5% CO_2_ in Dulbecco’s modified Eagle’s medium: Nutrient Mixture F-12 (F-12/DMEM (1:1), Gibco, USA) supplemented with 5% foetal bovine serum (FBS, Gibco, USA). For chondrogenesis induction, the medium was supplemented with 5% FBS, 1% insulin-transferrin-selenium (ITS), vitamin C (50 µg/ml) and ascorbate 2-phosphate (37.5 µg/ml).

### Cell transfection

Murine ATDC5 cells were allowed to reach 50–70% confluence at the time of transfection, and they were transfected with miRNA negative control, miR-20a mimic, miR-20a inhibitor (GenePharma, China) and ATG7 siRNA using Lipofectamine 2000 (Invitrogen, USA) for 24 h before experimental culture in accordance with the manufacturer’s protocol. The dose of each miR-20a-related reagent was 50 nM, and that of ATG7 siRNA (sense: GGAGUCACAGCUCUUCCUUTT, antisense: AAGGAAGAGCUGUGACUCCTT) was 50 pmol. The mRFP-GFP-LC3 adenovirus was purchased from HanBio Technology (Shanghai, China) and was used to transfect ATDC5 cells 48 h before induction culture at an MOI (multiplicity of infection) of 100 according to the manufacturer’s instructions to monitor autophagic flux.

### Quantitative real-time PCR

Total RNA from transfected cells was collected, placed in Trizol reagent (Invitrogen), homogenized for 20 seconds and incubated on ice for 5 min. Then, chloroform, isopropanol and absolute ethanol were sequentially added and centrifuged at 12000 r.p.m. after each addition for ten minutes at 4 °C. The precipitate was obtained and dried at room temperature for 10 min and then added to the reverse transcription reaction system. Quantitative real-time PCR (qRT-PCR) was performed to detect relative mRNAs using a PrimeScript™ RT Reagent Kit (Takara, Japan). For the miRNAs, cDNA was synthesized using a MiR-X™ miRNA First-Strand Synthesis Kit (Takara), and qRT-PCR was performed using a TB Green™ Kit (Takara). qRT-PCR was performed in triplicate using a LightCycler 480 II quantitative PCR system (Roche, Switzerland). A comparative Ct (2^−ΔΔCT^) method was used for analysis, and U6 and GAPDH served as internal reference genes. The primers for qRT-PCR are shown in Supplemental Table S1.

### Western blot analysis

For western blot analysis, cells were washed three times with PBS and then added to lysis buffer. Cell lysates were incubated on ice for 30 min; then, the soluble fraction was isolated by centrifugation at 12,000 r.p.m. for 10 min at 4 °C. Total protein concentrations of the cellular extracts were measured using a colourimetric BCA protein assay kit (Beyotime). Protein extracts were then loaded onto 10% SDS-PAGE gels and transferred onto a polyvinylidene difluoride membrane (PVDF, Millipore, USA). After blocking with 5% nonfat milk, the membranes were probed with primary antibodies at 4 °C with gentle shaking overnight. Next, the membranes were shaken at room temperature for 30 min and washed with TBST three times, followed by incubation with secondary antibodies at 37 °C for 1 h. Finally, after washing three times, the chemiluminescent signals on the membranes were detected using an ECL reagent (Thermo, USA). Densitometry values were normalized to the intensity of the corresponding bands for GAPDH. Quantitative analysis of western blotting was performed using Adobe Photoshop CS6 and ImageJ. The primary antibodies used in this study are listed in Supplemental Table S2.

### Fluorescence microscopy

ATDC5 cells were seeded onto slides and cultured for 12 h. Then, 6-Bio was used to treat the cells for 24 h, and an equal amount of DMSO was added to the control group. The cells were washed quickly with PBS 2 times, fixed with 4% paraformaldehyde in PBS for 20 min on ice and permeabilized with 0.5% Triton. The slides were then washed again with PBS, incubated with a blocking solution of 5% FBS for 1 h and exposed to an antibody against col2 at 4 °C overnight. After being washed three times with blocking solution, the slides were incubated with secondary antibody. Finally, the cells counterstained with DAPI were observed, and images were collected using a fluorescence microscope (Olympus IX73, Japan).

### Analysis of autophagic flux

After transfection with mRFP-GFP-LC3 adenoviral vectors for 48 h, ATDC5 cells underwent chondrogenic induction culture and were then collected and fixed with 4% paraformaldehyde in PBS for 30 min on ice. After washing with PBS three times and staining with DAPI, the cells were observed and recorded at four time points (24 h, 36 h, 48 h, 72 h) using confocal microscopy (Leica TCS SP8, Germany) to monitor autophagic flux. This assay is based on the different pH stabilities of green and red fluorescent proteins. When autophagosomes are transferred into the acidic conditions (pH < 5) of lysosomes, the fluorescent EGFP signal will be quenched, while the mRFP fluorescent signal will be sustained. Therefore, colocalization of both GFP and mRFP fluorescence indicates a compartment that has not fused with a lysosome, such as phagophore or autophagosome; a mRFP signal without GFP corresponds to an amphisome or autolysosome. In green and red images, autophagy flux is increased when the numbers of both yellow and red puncta are increased, while it is blocked when there are more yellow puncta with no change in the number of red puncta or when the numbers of both yellow and red puncta are decreased in cells^39,40^. Quantification of the numbers of red and yellow puncta per cell determines autophagic flux.

### Histochemistry assay

After 10 days of culture, cells were collected and stained. For alizarin red staining and quantitative analysis, the cells were rinsed with PBS, fixed in 4% paraformaldehyde for 15 min, washed with ddH_2_O three times, and then stained with Alizarin Red S Kit (Solarbio, China) for mineralized nodules. The cells were washed again before taking photos. After that, 10% cetylpyridinium chloride was used to dissolve the nodules, and the OD (optical density) was measured at 562 nm. For ALP staining and activity analysis, cells were rinsed with PBS and treated with 1% Triton X-100. The ALP activity of the cell lysates was tested with an ALP Activity Assay Kit (Beyotime, China). The protein concentrations were examined using an Enhanced BCA Protein Assay Kit (Beyotime) following the manufacturer’s instructions. For alcian blue staining and analysis, cells were also fixed in 4% paraformaldehyde for 10 min and then washed and stained with an Alcian Blue Staining Kit (ScienceCell, USA). After staining, the cells were washed with diH_2_O 3 times, and 6 mol/L guanidine hydrochloride was added and incubated for 2 h at room temperature. The OD was measured at 630 nm, and the cells were observed and captured under an inverted microscope (Olympus IX73, Japan).

### TEM

TEM was used to detect autophagosomes. ATDC5 cells were transfected with a miR-20a inhibitor, Atg7 siRNA or both and then cultured for eight days. The cells were then collected and fixed with 2.5% glutaraldehyde in 0.05 M sodium phosphate buffer. After washing at least 5 times, the samples were post-fixed in 1% w/v oso4 in 0.1 M sodium cacodylate buffer for 2 h, followed by dehydration with an increasing concentration gradient of ethanol and epon; then, the cells were embedded within pure Epon812. Next, the samples were cut into 50-nm sections and stained with 3% uranyl acetate and lead citrate. Images were acquired using a JEM-1230 electron microscope (JEOL, JAPAN) at 3000× and 6000× magnification. For each experimental group, at least 10 cellular cross-sections were scanned for the quantification of autophagosomes.

### Statistical analysis

All experiments were repeated three times. Student’s t-test was used to evaluate the statistical significance of the results (GraphPad Prism, La Jolla, CA, USA). All P-values represented 2-sided tests of statistical significance (*P < 0.05, **P < 0.01, ns = not significant).

## Supplementary information

Supplementary information

## Data Availability

The data that support the findings of this study are available from the corresponding author upon reasonable request.
