# Author Correction: miR-20a suppresses chondrogenic differentiation of ATDC5 cells by regulating Atg7

**DOI:** 10.1038/s41598-020-58140-1

**Published:** 2020-01-21

**Authors:** Rui Xu, Yuhao Wei, Xing Yin, Bing Shi, Jingtao Li

**Affiliations:** 1 State Key Laboratory of Oral Diseases & National Clinical Research Centre for Oral Diseases & Department of Oral and Maxillofacial Surgery, West China Hospital of Stomatology, Sichuan University, 14 Ren Min Nan Road, Chengdu, 610041 P.R. China; 2 State Key Laboratory of Oral Diseases & National Clinical Research Centre for Oral Diseases & Department of Orthodontics, West China Hospital of Stomatology, Sichuan University, 14 Ren Min Nan Road, Chengdu, 610041 P.R. China

Correction to: *Scientific Reports* 10.1038/s41598-019-45502-7, published online 25 June 2019

This Article contains errors in Figure 2F, where incorrect images are shown for the Alcian blue staining of the miR NC group, and the ALP staining of the Control group. The correct Figure 2 appears below as Figure 1.

**Figure 1:**
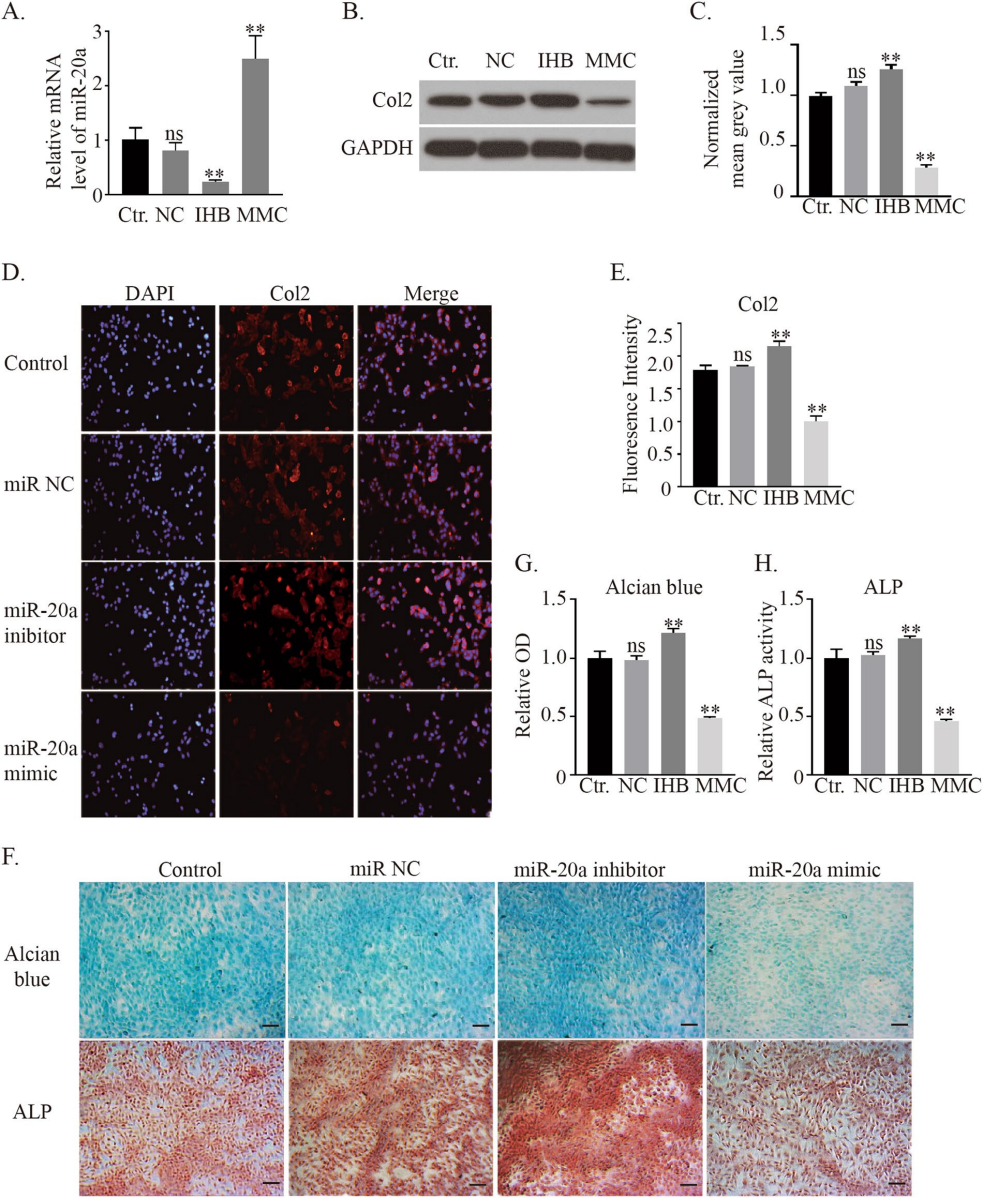
.

The conclusions of the Article are unaffected by these changes.

