# Supplementary material for: miR-20a suppresses chondrogenic differentiation of ATDC5 cells by regulating Atg7
Source: Sci Rep. 2019 Jun 25;9:9243. doi: 10.1038/s41598-019-45502-7 (PMC6592888; doi:10.1038/s41598-019-45502-7)
Supplement: Supplementary file 1 — Supplementary information [file 41598_2019_45502_MOESM1_ESM.pdf]

miR-20a suppresses chondrogenic differentiation of ATDC5 cells by regulating Atg7

Rui Xu[1], Yuhao Wei[1], Xing Yin[2], Bing Shi[1*], Jingtao Li[1,*]

1. State Key Laboratory of Oral Diseases & National Clinical Research Centre for Oral Diseases & Department of Oral and Maxillofacial Surgery, West China Hospital of Stomatology, Sichuan University, 14 Ren Min Nan Road, Chengdu, 610041, P. R. China.
2. State Key Laboratory of Oral Diseases & National Clinical Research Centre for Oral Diseases & Department of Orthodontics, West China Hospital of Stomatology, Sichuan University, 14 Ren Min Nan Road, Chengdu, 610041, P. R. China.

*Correspondence and requests for materials should be addressed to Jingtao. Li (lijingtao86@163.com) and Bing Shi (shibingcn@sina.com)

Table S1. The primers for qRT-PCR analysis

microRNA	Sequence
Mir universal primer	CCAGTCTCAGGGTCCGAGGTATTC
miR-17	TGCGGCCAAAGTGCTTACAGTGC
miR-18a	TGCGGCTAAGGTGCATCTAGTGC
miR-19a	TGCGGCTAGTTTTGCATAGTTG
miR-19b-1	TGCGGCAGTTTTGCAGGTTTGCA
miR-20a	TGCGGCTAAAGTGCTTATAGTGC

miR-92a-1	TGCGGCAGGTTGGGATTTGTCGC
U6-F	CTCGCTTCGGCAGCACA
U6-R	AACGCTTCACGAATTTGCGT
GAPDH-F	AGGACTGGATAAGCAGGGCG
GAPDH-R	CTGGAACAGGGAGGAGCAGA
Col2-F	CGGTCCTACGGTGTCAGGG
Col2-R	GGCAAGATGAGGGCTTCCATA
ATG7-F	CGGAAGTTGAGCGGCGAC
ATG7-R	GCAGGAAAGCAGTGTGGAGTT

Table S2. Antibodies used in this study.

ANTIBODIES	SOURCE	IDENTIFIER	Application
Anti-LC3B	Sigma-Aldrich	L7543	WB
Anti-Collagen II	Affinity	AF0135	WB, IF
Anti-p62	Abcam	ab155686	WB
Anti-ATG7	Abcam	ab133528	WB
Anti-Osteopontin	Abcam	ab93876	WB
Anti-p-Bcl2	Abcam	ab138406	WB
Anti-GAPDH	Abcam	ab8245	WB
